# 1-(4-Bromo­phen­yl)-2-{5-[(3,5-dimethyl-1*H*-pyrazol-1-yl)meth­yl]-4-phenyl-4*H*-1,2,4-triazol-3-ylsulfan­yl}ethanone

**DOI:** 10.1107/S1600536809000403

**Published:** 2009-01-10

**Authors:** Shan-Mei Xiao

**Affiliations:** a College of Materials Science and Chemical Engineering, Jinhua College of Profession and Technology, Jinhua, Zhejiang 321017, People’s Republic of China

## Abstract

The title compound, C_22_H_20_BrN_5_OS, is a potent new fungicide. The planes of the phenyl and pyrozole rings are almost perpendicular, making a dihedral angle of 86.5 (4)°. There are two non-classical inter­molecular C—H⋯O and C—H⋯N hydrogen bonds in the crystal structure.

## Related literature

For background to heterocyclic compounds, see: Gong *et al.* (2008 ▶); Liu *et al.* (2007 ▶). For the synthesis, see: He *et al.* (2008 ▶).
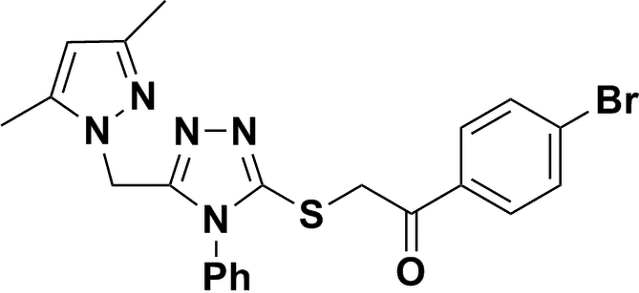


## Experimental

### 

#### Crystal data

C_22_H_20_BrN_5_OS
*M* *_r_* = 482.40
Triclinic, 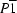

*a* = 8.705 (2) Å
*b* = 9.173 (2) Å
*c* = 14.564 (4) Å
α = 94.561 (4)°
β = 97.659 (4)°
γ = 103.086 (4)°
*V* = 1115.3 (5) Å^3^
*Z* = 2
Mo *K*α radiation
μ = 1.96 mm^−1^
*T* = 294 (2) K
0.28 × 0.24 × 0.20 mm

#### Data collection

Bruker SMART CCD area-detector diffractometer
Absorption correction: multi-scan (*SADABS*; Sheldrick, 1996 ▶) *T*_min_ = 0.592, *T*_max_ = 0.674
5704 measured reflections
3914 independent reflections
2419 reflections with *I* > 2σ(*I*)
*R*_int_ = 0.021

#### Refinement

*R*[*F* ^2^ > 2σ(*F* ^2^)] = 0.041
*wR*(*F* ^2^) = 0.101
*S* = 1.02
3914 reflections
273 parameters
H-atom parameters constrained
Δρ_max_ = 0.39 e Å^−3^
Δρ_min_ = −0.37 e Å^−3^

### 

Data collection: *SMART* (Bruker, 2004 ▶); cell refinement: *SAINT* (Bruker, 2004 ▶); data reduction: *SAINT*; program(s) used to solve structure: *SHELXS97* (Sheldrick, 2008 ▶); program(s) used to refine structure: *SHELXL97* (Sheldrick, 2008 ▶); molecular graphics: *SHELXTL* (Sheldrick, 2008 ▶); software used to prepare material for publication: *SHELXTL*.

## Supplementary Material

Crystal structure: contains datablocks global, I. DOI: 10.1107/S1600536809000403/bq2115sup1.cif

Structure factors: contains datablocks I. DOI: 10.1107/S1600536809000403/bq2115Isup2.hkl

Additional supplementary materials:  crystallographic information; 3D view; checkCIF report

## Figures and Tables

**Table 1:** Hydrogen-bond geometry (Å, °)

*D*—H⋯*A*	*D*—H	H⋯*A*	*D*⋯*A*	*D*—H⋯*A*
C6—H6*B*⋯O1^i^	0.97	2.45	3.365 (3)	157
C15—H15*B*⋯N4^ii^	0.97	2.50	3.429 (3)	161

Symmetry codes: (i) 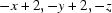; (ii) 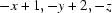.

**Fig. 1.**
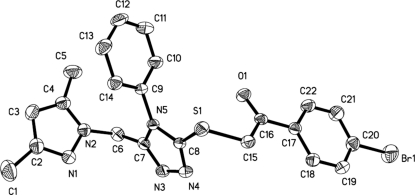
The structure of (I) with displacement ellipsoids drawn at the 30% probability level.

**Fig. 2.**
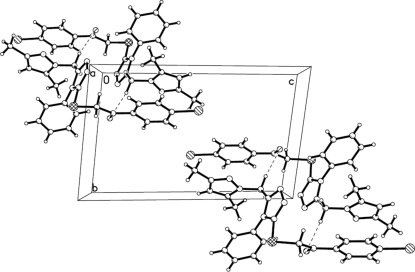
Partial packing diagram for (I). The dotted lines show the C—H–O bond.

**Fig. 3.**
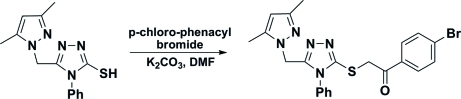
The formation of the title compound.

## supplementary crystallographic information

### Comment

A variety of pyrazole and triazole heterocyclics could exhibit many activities. Meanwhile, heterocyclic compounds is an important developmental direction in medical (Gong *et al.*, 2008) and pesticidal (Liu *et al.*, 2007) chemistry.

In view of these facts and in continuation of our interest in the agriculture, we attempted to synthesize a series of amide derivatives, some of which have comparatively high fungicidal activity.

The molecular structure of title compound is showing in Fig.1. The x-ray analysis reveals that acetyl group is a planar with thio-ether group. The pyrozole ring is vertical with the benzene ring [dihedral angle 93.5 (4)°]. The packing of the structure is due to the weak intermolecular C-H..O and C-H..N H-bonds (Table 1. and Fig 2.).

### Experimental

The compound 5-((3,5-Dimethyl-1*H*-pyrazol-1-yl)methyl)-4-phenyl-4*H*-1,2,4 -triazole-3-thiol was synthesized according to the reference (He *et al.*, 2008). Then added *p*-bromo-phenacyl bromide, potassium carbonate anhydrous and *N*,*N*-Dimethyl formamide was stirred at room temperature for 5 h, giving the title compound. Colorless single crystals suitable for x-ray diffraction were obtained by recrystallization from a mixture of ethyl acetate and petroleum ether.

### Refinement

The H atoms bonded to C and N atoms were positioned geometrically and refined using a riding model [aromatic C—H=0.93 Å, aliphatic C—H = 0.97 (2) Å, N—H=0.86 Å, *U*_iso_(H) = 1.2*U*_eq_(C)].

### Crystal data

**Table:**

C_22_H_20_BrN_5_OS	*Z* = 2
*M**_r_* = 482.40	*F*(000) = 492
Triclinic, *P*1	*D*_x_ = 1.436 Mg m^−^^3^
Hall symbol: -P 1	Mo *K*α radiation, λ = 0.71073 Å
*a* = 8.705 (2) Å	Cell parameters from 1669 reflections
*b* = 9.173 (2) Å	θ = 2.6–23.0°
*c* = 14.564 (4) Å	µ = 1.96 mm^−^^1^
α = 94.561 (4)°	*T* = 294 K
β = 97.659 (4)°	Rhombic, colorless
γ = 103.086 (4)°	0.28 × 0.24 × 0.20 mm
*V* = 1115.3 (5) Å^3^	

### Data collection

**Table:**

Bruker SMART CCD area-detector diffractometer	3914 independent reflections
Radiation source: fine-focus sealed tube	2419 reflections with *I* > 2σ(*I*)
graphite	*R*_int_ = 0.021
φ and ω scans	θ_max_ = 25.0°, θ_min_ = 1.4°
Absorption correction: multi-scan (*SADABS*; Sheldrick, 1996)	*h* = −10→10
*T*_min_ = 0.592, *T*_max_ = 0.674	*k* = −10→10
5704 measured reflections	*l* = −17→6

### Refinement

**Table:**

Refinement on *F*^2^	Primary atom site location: structure-invariant direct methods
Least-squares matrix: full	Secondary atom site location: difference Fourier map
*R*[*F*^2^ > 2σ(*F*^2^)] = 0.041	Hydrogen site location: inferred from neighbouring sites
*wR*(*F*^2^) = 0.101	H-atom parameters constrained
*S* = 1.02	*w* = 1/[σ^2^(*F*_o_^2^) + (0.0351*P*)^2^ + 0.4689*P*] where *P* = (*F*_o_^2^ + 2*F*_c_^2^)/3
3914 reflections	(Δ/σ)_max_ = 0.001
273 parameters	Δρ_max_ = 0.39 e Å^−^^3^
0 restraints	Δρ_min_ = −0.37 e Å^−^^3^

### Special details

**Table:**

Geometry. All e.s.d.'s (except the e.s.d. in the dihedral angle between two l.s. planes) are estimated using the full covariance matrix. The cell e.s.d.'s are taken into account individually in the estimation of e.s.d.'s in distances, angles and torsion angles; correlations between e.s.d.'s in cell parameters are only used when they are defined by crystal symmetry. An approximate (isotropic) treatment of cell e.s.d.'s is used for estimating e.s.d.'s involving l.s. planes.
Refinement. Refinement of *F*^2^ against ALL reflections. The weighted *R*-factor *wR* and goodness of fit *S* are based on *F*^2^, conventional *R*-factors *R* are based on *F*, with *F* set to zero for negative *F*^2^. The threshold expression of *F*^2^ > σ(*F*^2^) is used only for calculating *R*-factors(gt) *etc*. and is not relevant to the choice of reflections for refinement. *R*-factors based on *F*^2^ are statistically about twice as large as those based on *F*, and *R*- factors based on ALL data will be even larger.

### Fractional atomic coordinates and isotropic or equivalent isotropic displacement parameters (Å^2^)

**Table:**

	*x*	*y*	*z*	*U*_iso_*/*U*_eq_	
Br1	0.29289 (6)	0.67651 (6)	−0.50744 (3)	0.0905 (2)	
S1	0.55698 (11)	0.71293 (10)	0.09603 (6)	0.0490 (3)	
O1	0.6651 (3)	0.6235 (2)	−0.07550 (16)	0.0513 (6)	
N1	1.0999 (3)	1.1534 (3)	0.3101 (2)	0.0510 (7)	
N2	1.1688 (3)	1.0707 (3)	0.25343 (19)	0.0454 (7)	
N3	0.8643 (4)	1.0825 (3)	0.07347 (19)	0.0501 (7)	
N4	0.7096 (3)	0.9911 (3)	0.05655 (19)	0.0499 (7)	
N5	0.8674 (3)	0.8715 (3)	0.13335 (16)	0.0364 (6)	
C1	1.1192 (6)	1.2185 (5)	0.4789 (3)	0.0941 (15)	
H1A	1.0703	1.2973	0.4597	0.141*	
H1B	1.2134	1.2611	0.5236	0.141*	
H1C	1.0455	1.1474	0.5066	0.141*	
C2	1.1639 (5)	1.1397 (4)	0.3953 (3)	0.0590 (10)	
C3	1.2727 (5)	1.0505 (4)	0.3942 (3)	0.0640 (11)	
H3	1.3327	1.0254	0.4456	0.077*	
C4	1.2744 (4)	1.0070 (4)	0.3029 (3)	0.0548 (10)	
C5	1.3679 (5)	0.9113 (5)	0.2587 (3)	0.0835 (14)	
H5A	1.2977	0.8172	0.2309	0.125*	
H5B	1.4481	0.8926	0.3052	0.125*	
H5C	1.4182	0.9623	0.2115	0.125*	
C6	1.1288 (4)	1.0667 (4)	0.1533 (2)	0.0507 (9)	
H6A	1.1863	1.0030	0.1228	0.061*	
H6B	1.1632	1.1676	0.1360	0.061*	
C7	0.9553 (4)	1.0093 (3)	0.1199 (2)	0.0405 (8)	
C8	0.7156 (4)	0.8664 (3)	0.0931 (2)	0.0398 (8)	
C9	0.9262 (4)	0.7495 (3)	0.1701 (2)	0.0363 (7)	
C10	0.9932 (4)	0.6635 (4)	0.1140 (3)	0.0571 (10)	
H10	1.0027	0.6845	0.0533	0.069*	
C11	1.0469 (5)	0.5443 (4)	0.1486 (3)	0.0730 (12)	
H11	1.0928	0.4846	0.1112	0.088*	
C12	1.0322 (5)	0.5149 (4)	0.2383 (3)	0.0658 (11)	
H12	1.0665	0.4340	0.2613	0.079*	
C13	0.9675 (5)	0.6034 (4)	0.2940 (3)	0.0635 (11)	
H13	0.9603	0.5840	0.3552	0.076*	
C14	0.9126 (4)	0.7216 (4)	0.2602 (2)	0.0514 (9)	
H14	0.8671	0.7813	0.2978	0.062*	
C15	0.4513 (4)	0.7061 (4)	−0.0201 (2)	0.0461 (9)	
H15A	0.3481	0.6353	−0.0262	0.055*	
H15B	0.4326	0.8047	−0.0291	0.055*	
C16	0.5408 (4)	0.6597 (3)	−0.0953 (2)	0.0395 (8)	
C17	0.4720 (4)	0.6607 (3)	−0.1940 (2)	0.0420 (8)	
C18	0.3491 (4)	0.7294 (4)	−0.2204 (2)	0.0509 (9)	
H18	0.3023	0.7726	−0.1749	0.061*	
C19	0.2954 (4)	0.7345 (4)	−0.3134 (3)	0.0587 (10)	
H19	0.2140	0.7820	−0.3308	0.070*	
C20	0.3635 (5)	0.6689 (4)	−0.3794 (2)	0.0557 (10)	
C21	0.4837 (5)	0.5963 (4)	−0.3557 (3)	0.0638 (11)	
H21	0.5276	0.5505	−0.4015	0.077*	
C22	0.5363 (4)	0.5937 (4)	−0.2631 (3)	0.0547 (10)	
H22	0.6173	0.5457	−0.2463	0.066*	

### Atomic displacement parameters (Å^2^)

**Table:**

	*U*^11^	*U*^22^	*U*^33^	*U*^12^	*U*^13^	*U*^23^
Br1	0.1110 (4)	0.1126 (4)	0.0534 (3)	0.0427 (3)	0.0014 (3)	0.0149 (3)
S1	0.0501 (6)	0.0487 (5)	0.0493 (5)	0.0125 (4)	0.0070 (4)	0.0112 (4)
O1	0.0395 (14)	0.0570 (15)	0.0614 (15)	0.0204 (12)	0.0066 (12)	0.0070 (12)
N1	0.0537 (19)	0.0510 (18)	0.0496 (19)	0.0163 (15)	0.0084 (15)	0.0027 (15)
N2	0.0429 (17)	0.0426 (16)	0.0494 (18)	0.0079 (14)	0.0068 (14)	0.0046 (14)
N3	0.067 (2)	0.0359 (16)	0.0486 (17)	0.0156 (16)	0.0042 (16)	0.0102 (14)
N4	0.058 (2)	0.0389 (17)	0.0548 (18)	0.0192 (16)	0.0013 (15)	0.0076 (14)
N5	0.0454 (17)	0.0327 (15)	0.0344 (15)	0.0150 (13)	0.0064 (13)	0.0061 (12)
C1	0.111 (4)	0.114 (4)	0.057 (3)	0.031 (3)	0.015 (3)	−0.008 (3)
C2	0.063 (3)	0.059 (2)	0.052 (2)	0.011 (2)	0.007 (2)	0.005 (2)
C3	0.068 (3)	0.059 (3)	0.058 (3)	0.013 (2)	−0.010 (2)	0.011 (2)
C4	0.045 (2)	0.043 (2)	0.074 (3)	0.0081 (18)	0.004 (2)	0.005 (2)
C5	0.067 (3)	0.070 (3)	0.117 (4)	0.033 (2)	0.003 (3)	−0.001 (3)
C6	0.057 (2)	0.048 (2)	0.047 (2)	0.0074 (18)	0.0164 (18)	0.0065 (17)
C7	0.050 (2)	0.0356 (19)	0.0362 (18)	0.0082 (17)	0.0104 (16)	0.0041 (15)
C8	0.051 (2)	0.0351 (19)	0.0361 (18)	0.0185 (17)	0.0032 (16)	0.0019 (15)
C9	0.0392 (19)	0.0319 (17)	0.0402 (19)	0.0122 (15)	0.0063 (15)	0.0078 (15)
C10	0.072 (3)	0.056 (2)	0.052 (2)	0.033 (2)	0.012 (2)	0.0057 (19)
C11	0.085 (3)	0.059 (3)	0.085 (3)	0.043 (2)	0.010 (3)	0.000 (2)
C12	0.064 (3)	0.048 (2)	0.087 (3)	0.023 (2)	−0.005 (2)	0.024 (2)
C13	0.076 (3)	0.063 (3)	0.055 (2)	0.020 (2)	0.006 (2)	0.027 (2)
C14	0.062 (2)	0.054 (2)	0.044 (2)	0.0220 (19)	0.0124 (18)	0.0119 (17)
C15	0.044 (2)	0.043 (2)	0.053 (2)	0.0147 (17)	0.0041 (17)	0.0054 (16)
C16	0.036 (2)	0.0261 (17)	0.055 (2)	0.0050 (15)	0.0059 (17)	0.0036 (15)
C17	0.040 (2)	0.0376 (19)	0.048 (2)	0.0095 (16)	0.0066 (17)	0.0050 (16)
C18	0.050 (2)	0.054 (2)	0.051 (2)	0.0198 (19)	0.0067 (18)	−0.0005 (18)
C19	0.058 (2)	0.059 (2)	0.063 (3)	0.027 (2)	−0.002 (2)	0.007 (2)
C20	0.061 (2)	0.058 (2)	0.048 (2)	0.013 (2)	0.006 (2)	0.0078 (19)
C21	0.069 (3)	0.077 (3)	0.052 (2)	0.029 (2)	0.014 (2)	0.002 (2)
C22	0.054 (2)	0.060 (2)	0.057 (2)	0.027 (2)	0.0087 (19)	0.0051 (19)

### Geometric parameters (Å, °)

**Table:**

Br1—C20	1.896 (3)	C6—H6B	0.9700
S1—C8	1.742 (3)	C9—C10	1.365 (4)
S1—C15	1.803 (3)	C9—C14	1.372 (4)
O1—C16	1.208 (3)	C10—C11	1.387 (5)
N1—C2	1.319 (4)	C10—H10	0.9300
N1—N2	1.362 (4)	C11—C12	1.371 (5)
N2—C4	1.356 (4)	C11—H11	0.9300
N2—C6	1.449 (4)	C12—C13	1.363 (5)
N3—C7	1.306 (4)	C12—H12	0.9300
N3—N4	1.395 (4)	C13—C14	1.380 (5)
N4—C8	1.308 (4)	C13—H13	0.9300
N5—C8	1.361 (4)	C14—H14	0.9300
N5—C7	1.365 (4)	C15—C16	1.514 (4)
N5—C9	1.443 (4)	C15—H15A	0.9700
C1—C2	1.506 (5)	C15—H15B	0.9700
C1—H1A	0.9600	C16—C17	1.484 (4)
C1—H1B	0.9600	C17—C22	1.382 (4)
C1—H1C	0.9600	C17—C18	1.387 (4)
C2—C3	1.385 (5)	C18—C19	1.381 (5)
C3—C4	1.360 (5)	C18—H18	0.9300
C3—H3	0.9300	C19—C20	1.363 (5)
C4—C5	1.491 (5)	C19—H19	0.9300
C5—H5A	0.9600	C20—C21	1.382 (5)
C5—H5B	0.9600	C21—C22	1.369 (5)
C5—H5C	0.9600	C21—H21	0.9300
C6—C7	1.483 (5)	C22—H22	0.9300
C6—H6A	0.9700		
			
C8—S1—C15	98.77 (15)	C10—C9—N5	119.1 (3)
C2—N1—N2	104.6 (3)	C14—C9—N5	119.5 (3)
C4—N2—N1	111.8 (3)	C9—C10—C11	119.2 (3)
C4—N2—C6	129.2 (3)	C9—C10—H10	120.4
N1—N2—C6	118.9 (3)	C11—C10—H10	120.4
C7—N3—N4	107.4 (3)	C12—C11—C10	119.7 (4)
C8—N4—N3	106.9 (3)	C12—C11—H11	120.1
C8—N5—C7	105.1 (2)	C10—C11—H11	120.1
C8—N5—C9	127.1 (3)	C13—C12—C11	120.4 (3)
C7—N5—C9	127.3 (3)	C13—C12—H12	119.8
C2—C1—H1A	109.5	C11—C12—H12	119.8
C2—C1—H1B	109.5	C12—C13—C14	120.4 (4)
H1A—C1—H1B	109.5	C12—C13—H13	119.8
C2—C1—H1C	109.5	C14—C13—H13	119.8
H1A—C1—H1C	109.5	C9—C14—C13	118.9 (3)
H1B—C1—H1C	109.5	C9—C14—H14	120.6
N1—C2—C3	111.3 (3)	C13—C14—H14	120.6
N1—C2—C1	120.7 (4)	C16—C15—S1	112.9 (2)
C3—C2—C1	127.9 (4)	C16—C15—H15A	109.0
C4—C3—C2	106.4 (3)	S1—C15—H15A	109.0
C4—C3—H3	126.8	C16—C15—H15B	109.0
C2—C3—H3	126.8	S1—C15—H15B	109.0
N2—C4—C3	105.9 (3)	H15A—C15—H15B	107.8
N2—C4—C5	123.3 (4)	O1—C16—C17	120.9 (3)
C3—C4—C5	130.9 (4)	O1—C16—C15	121.0 (3)
C4—C5—H5A	109.5	C17—C16—C15	118.1 (3)
C4—C5—H5B	109.5	C22—C17—C18	118.4 (3)
H5A—C5—H5B	109.5	C22—C17—C16	118.5 (3)
C4—C5—H5C	109.5	C18—C17—C16	123.1 (3)
H5A—C5—H5C	109.5	C19—C18—C17	120.7 (3)
H5B—C5—H5C	109.5	C19—C18—H18	119.6
N2—C6—C7	112.5 (3)	C17—C18—H18	119.6
N2—C6—H6A	109.1	C20—C19—C18	119.0 (3)
C7—C6—H6A	109.1	C20—C19—H19	120.5
N2—C6—H6B	109.1	C18—C19—H19	120.5
C7—C6—H6B	109.1	C19—C20—C21	121.8 (3)
H6A—C6—H6B	107.8	C19—C20—Br1	119.6 (3)
N3—C7—N5	110.1 (3)	C21—C20—Br1	118.6 (3)
N3—C7—C6	125.2 (3)	C22—C21—C20	118.3 (3)
N5—C7—C6	124.7 (3)	C22—C21—H21	120.9
N4—C8—N5	110.5 (3)	C20—C21—H21	120.9
N4—C8—S1	127.3 (3)	C21—C22—C17	121.7 (3)
N5—C8—S1	122.2 (2)	C21—C22—H22	119.1
C10—C9—C14	121.4 (3)	C17—C22—H22	119.1
			
C2—N1—N2—C4	−0.2 (4)	C15—S1—C8—N5	−141.3 (3)
C2—N1—N2—C6	−177.5 (3)	C8—N5—C9—C10	93.5 (4)
C7—N3—N4—C8	−0.5 (3)	C7—N5—C9—C10	−76.7 (4)
N2—N1—C2—C3	0.3 (4)	C8—N5—C9—C14	−86.0 (4)
N2—N1—C2—C1	179.0 (3)	C7—N5—C9—C14	103.8 (4)
N1—C2—C3—C4	−0.2 (5)	C14—C9—C10—C11	0.7 (5)
C1—C2—C3—C4	−178.9 (4)	N5—C9—C10—C11	−178.7 (3)
N1—N2—C4—C3	0.0 (4)	C9—C10—C11—C12	−0.1 (6)
C6—N2—C4—C3	177.0 (3)	C10—C11—C12—C13	−1.1 (6)
N1—N2—C4—C5	−179.6 (3)	C11—C12—C13—C14	1.5 (6)
C6—N2—C4—C5	−2.6 (5)	C10—C9—C14—C13	−0.3 (5)
C2—C3—C4—N2	0.1 (4)	N5—C9—C14—C13	179.2 (3)
C2—C3—C4—C5	179.7 (4)	C12—C13—C14—C9	−0.9 (6)
C4—N2—C6—C7	124.9 (4)	C8—S1—C15—C16	68.2 (2)
N1—N2—C6—C7	−58.3 (4)	S1—C15—C16—O1	4.1 (4)
N4—N3—C7—N5	0.9 (3)	S1—C15—C16—C17	−175.6 (2)
N4—N3—C7—C6	−179.1 (3)	O1—C16—C17—C22	11.7 (5)
C8—N5—C7—N3	−0.9 (3)	C15—C16—C17—C22	−168.6 (3)
C9—N5—C7—N3	171.0 (3)	O1—C16—C17—C18	−166.4 (3)
C8—N5—C7—C6	179.0 (3)	C15—C16—C17—C18	13.2 (4)
C9—N5—C7—C6	−9.0 (5)	C22—C17—C18—C19	−1.8 (5)
N2—C6—C7—N3	122.0 (3)	C16—C17—C18—C19	176.3 (3)
N2—C6—C7—N5	−58.0 (4)	C17—C18—C19—C20	0.9 (5)
N3—N4—C8—N5	−0.1 (3)	C18—C19—C20—C21	0.6 (6)
N3—N4—C8—S1	178.2 (2)	C18—C19—C20—Br1	−179.3 (3)
C7—N5—C8—N4	0.6 (3)	C19—C20—C21—C22	−1.2 (6)
C9—N5—C8—N4	−171.4 (3)	Br1—C20—C21—C22	178.8 (3)
C7—N5—C8—S1	−177.8 (2)	C20—C21—C22—C17	0.3 (6)
C9—N5—C8—S1	10.3 (4)	C18—C17—C22—C21	1.2 (5)
C15—S1—C8—N4	40.6 (3)	C16—C17—C22—C21	−177.0 (3)

### Hydrogen-bond geometry (Å, °)

**Table:**

*D*—H···*A*	*D*—H	H···*A*	*D*···*A*	*D*—H···*A*
C6—H6B···O1^i^	0.97	2.45	3.365 (3)	157
C15—H15B···N4^ii^	0.97	2.50	3.429 (3)	161

Symmetry codes: (i) −*x*+2, −*y*+2, −*z*; (ii) −*x*+1, −*y*+2, −*z*.
